# Dietary organic acids ameliorate high stocking density stress-induced intestinal inflammation through the restoration of intestinal microbiota in broilers

**DOI:** 10.1186/s40104-022-00776-2

**Published:** 2022-11-14

**Authors:** Dong Dai, Guanghai Qi, Jing Wang, Haijun Zhang, Kai Qiu, Yanming Han, Yuanyuan Wu, Shugeng Wu

**Affiliations:** 1 Laboratory of Quality and Safety Risk Assessment for Animal Products on Feed Hazards (Beijing) of the Ministry of Agriculture and Rural Affairs, Institute of Feed Research, Chinese Academy of Agricultural Sciences, No. 12 Zhongguancun South St., Haidian district, Beijing, China; 2 Trouw Nutrition Research & Development Centers, Amersfoort, Netherlands

**Keywords:** Broiler, High stocking density, Intestinal inflammation, Intestinal microbiota, Organic acid, Short-chain fatty acid

## Abstract

**Background:**

High stocking density (HSD) stress has detrimental effects on growth performance, intestinal barrier function, and intestinal microbiota in intensive animal production. Organic acids (OA) are widely used as feed additives for their ability to improve growth performance and intestinal health in poultry. However, whether dietary OA can ameliorate HSD stress-induced impaired intestinal barrier in broilers remains elusive. In this study, a total of 528 one-day-old male Arbor Acres broilers were randomly allocated into 3 treatments with 12 replicates per treatment including 10 birds for normal stocking density and 17 birds for HSD. The dietary treatments were as follows: 1) Normal stocking density + basal diet; 2) HSD + basal diets; 3) HSD + OA.

**Results:**

HSD stress can induce increased levels of serum corticosterone, lipopolysaccharides, interleukin-1β, tumor necrosis factor-α, and down-regulated mRNA expression of *ZO-1*, resulting in compromised growth performance of broilers (*P* < 0.05). Dietary OA could significantly reduce levels of serum corticosterone, lipopolysaccharides, interleukin-1β, and tumor necrosis factor-α, which were accompanied by up-regulated interleukin-10, mRNA expression of *ZO-1*, and growth performance (*P* < 0.05). Moreover, OA could down-regulate the mRNA expression of *TLR4* and *MyD88* to inhibit the NF-κB signaling pathway (*P* < 0.05). Additionally, HSD stress significantly decreased the abundance of Bacteroidetes and disturbed the balance of microbial ecosystems, whereas OA significantly increased the abundance of Bacteroidetes and restored the disordered gut microbiota by reducing competitive and exploitative interactions in microbial communities (*P* < 0.05). Meanwhile, OA significantly increased the content of acetic and butyric acids, which showed significant correlations with intestinal inflammation indicators (*P* < 0.05).

**Conclusions:**

Dietary OA ameliorated intestinal inflammation and growth performance of broilers through restoring the disordered gut microbial compositions and interactions induced by HSD and elevating short-chain fatty acid production to inhibit the TLR4/NF-κB signaling pathway. These findings demonstrated the critical role of intestinal microbiota in mediating the HSD-induced inflammatory responses, contributing to exploring nutritional strategies to alleviate HSD-induced stress in animals.

**Supplementary Information:**

The online version contains supplementary material available at 10.1186/s40104-022-00776-2.

## Introduction

Global chicken meat production has been growing in recent decades and reaches 99.10 million tons in 2021 [1]. To meet market supply and achieve high efficiency, the high stocking density (HSD) rearing is commonly applied in broiler production to maximize the area of the cage [2]. The HSD is defined based on the number of chicks per square meter as well as the weight of birds per square meter in broiler production. Generally, a maximum stocking density of 33 kg/m^2^ or 16 chicks/m^2^ is acceptable as long as proper rearing management [3]. However, if stocking density exceeds the proper range, adverse effects on growth performance and intestinal health will be induced by HSD in broilers [4–6]. HSD stress can trigger corticosterone secretion and lead to metabolic disorders in broilers [7–11], causing decreased growth performance. Meanwhile, HSD exposure has detrimental effects on the intestinal barrier in terms of gene expressions of claudin-1, occludin, and zonula occludens-1 (*ZO-1*) [5, 12–14], which were often accompanied by increased lipopolysaccharides (LPS) level [15]. As a marker of intestinal barrier damage, LPS can bind to toll-like receptor 4 (TLR4) to stimulate the downstream signaling molecule and promote phosphorylation of NF-κB (p-NF-κB), thereby activating the TLR4/NF-κB signaling pathway to promote transcription of pro-inflammatory cytokines like IL-1β and TNF-α [16]. However, whether the impaired barrier function induced by HSD stress is related to the activation of the TLR4/NF-κB signaling pathway in broilers remains to be verified.

Notably, the LPS is composed of lipids and polysaccharides in the outer membrane of the cell wall of Gram-negative bacteria [17]. Previous studies have found alterations in the intestinal microbiota of broilers exposed to HSD stress [4, 18, 19]. It is well-known that the intestinal microbiota of poultry plays key roles in digestion, barrier, and immune function contributing to the improvement in growth performance [20, 21]. However, whether HSD stress-induced intestinal barrier damage is related to the intestinal microbiota of broilers remains elusive. Generally, the intestinal microbiota is involved in regulating immune responses of the host through short-chain fatty acids (SCFAs) produced by the fermentation of carbohydrates [22]. Numerous studies showed that SCFAs inhibited the activation of the TLR4/NF-κB signaling pathway and suppressed the generation of pro-inflammatory cytokines [23–25]. Thus, intestinal microbiota may act as a potential target for improving HSD stress-induced intestinal inflammation through nutritional interventions.

Organic acids (OA) are considered to be organic carboxylic acids with R-COOH, which are widely used in poultry as feed additives for their ability to improve growth performance and intestinal health [26–28]. OA can reduce the intestinal lumen pH and increase digestive enzyme activity to improve the apparent digestibility [29, 30]. In particular, OA can alter the intestinal microbiota of broilers due to its antimicrobial action via perforating semipermeable peptidoglycan or phospholipid membrane, then the dissociation and release of hydrogen ions reduce the pH to induced bacterial collapse [26, 31, 32]. Meanwhile, accumulating evidence implies that OA improves the intestinal barrier and immune function by shifting the intestinal microbiota of broilers [26, 33, 34]. However, whether dietary OA can ameliorate HSD stress-induced impaired intestinal barrier and compromised growth performance of broilers remains unknown.

It was hypothesized that dietary OA might shape the gut microbial compositions and interactions to ameliorate HSD stress-induced intestinal inflammation in broilers. The present study was designed to determine effects of dietary OA on growth performance, stress parameters, intestinal development, inflammatory responses, and compositions and interactions of intestinal microbiota in broilers exposed to the HSD. Subsequently, the intestinal SCFA profile and its associations with intestinal inflammation were analyzed to further explore microbial roles in regulating inflammatory responses. This study may facilitate a better understanding of intestinal microbiota and host crosstalk in the HSD-induced intestinal inflammation, contributing to optimizing nutritional strategies to alleviate HSD-induced stress in animals.

## Materials and methods

### Animal management and experimental treatments

A total of 528 one-day-old male Arbor Acres broilers were randomly allocated into 3 treatments with 12 replicates per treatment including 10 birds for normal stocking density (11 birds/m^2^ or 26.51 kg/m^2^) and 17 birds for high stocking density (18 birds/m^2^ or 43.38 kg/m^2^). The stocking density used in this study refers to previous studies [4, 35]. Experimental treatments were offered until 42 days of age as follows: 1) Normal stocking density fed basal diet (Negative control, NC); 2) High stocking density fed basal diets (Positive control, PC); 3) High stocking density fed basal diets supplemented with organic acids (Organic acids, OA). The basal diet was formulated to meet or exceed National Research Council [36] guidelines (Additional file 1: Table S1). The OA feed additive is a blend of free and buffered short-chain fatty acids (formic acid ≥ 12%, ammonium formate ≥ 12%, acetic acid ≥ 4.4%, propionic acid ≥ 5.2%, sorbic acid ≥ 0.4%, lactic acid ≥ 0.5 %, and citric acid ≥ 0.1%) combined with medium-chain fatty acids (caproic acid, caprylic acid, decanoic acid, and lauric acid) ≥ 8.6% on a silica carrier included in broiler feed at 2 g/kg (d 0–21) and 1 g/kg (d 22–42). Diet and water were supplied ad libitum in pellet form and by nipple drinkers, respectively. Broiler management was followed as the guideline for raising Arbor Acres broilers.

### Growth performance and litter quality and breast blister score

The body weight (BW), average daily gain (ADG), average daily feed intake (ADFI), and feed conversion ratio (feed:gain, F/G), was recorded at the phases of d 1–21, d 22–42, and d 1–42. On d 21 and 42. The litter of each pen will be collected and scored on a scale of 1 (normal) to 4 (diarrhea) as previously described [37]. Additionally, one bird will be randomly selected from each replicate pen, breast blister will be scored on both breasts and calculated to an average. The evaluation stand is as below: 1 score with normal no blister; 2 score with small range blister (0–2 cm^2^); 3 score with middle size blister (2–5 cm^2^); 4 score with large size blister (> 5 cm^2^).

### Determination of serum biochemical parameters

On d 42, one healthy bird with average body weight will be selected from each replicate and the blood will be taken through the wing vein. Blood samples were collected into tubes without anticoagulants and then centrifuged at 3000×*g* for 15 min at 4 °C to obtain serum and stored at − 80 °C. The contents of aspartate transaminase (AST), glucose (GLU), triglycerides (TG), total cholesterol (TC), high-density lipoprotein cholesterol (HDL-C), and low-density lipoprotein cholesterol (LDL-C) were measured using the KHB400 automatic biochemical analyzer (Kehua Bioengineering, Co. Ltd., Shanghai, China).

### Histological analysis and immunohistochemistry

The jejunum tissues were fixed in 4% paraformaldehyde solution for 24 h and then were stained with hematoxylin and eosin. The villus height and crypt depth were determined using an image processing and analyzing system (Inverted microscope: NIKON CI-S, Tokyo, Japan; Imaging system: NIKON DS-U3, Tokyo, Japan). Additionally, goblet cells were the major source of mucin and were studied by jejunum tissues stained with Alcian Blue and Periodic Acid-Schiff kit (Sigma Chemical Co., St Louis, MO, USA). All the sections were examined for the number of goblet cells of the villi using an Olympus DP12 CCD digital camera and Image-Pro Plus 6.0 software (Media Cybernetics, Bethesda, MD, USA). Moreover, jejunal paraffin sections were immunohistochemical staining to elucidate the subcellular localization of p-NF-κB and to evaluate the inflammatory injury degree, which was carried out according to the previous study [38].

### Enzyme-linked immunosorbent assay

Enzyme-linked immunosorbent assay is a simple, rapid, and accurate immunochemical assay based on the use of solid-phase adsorption in combination with immunoenzyme technology for the quantitative detection of corresponding antigens or antibodies. Therefore, we used an enzyme-linked immunosorbent assay to detect levels of inflammatory cytokines, further demonstrating the HSD-induced stress and inflammatory response in broilers. Jejunal mucosa tissues were homogenized in ice-cold PBS and centrifuged at 1000 r/min at 4 °C for 20 min. The supernatant was collected for the chicken enzyme-linked immunosorbent assay kit (Shanghai Enzyme-linked Biotechnology Co., Ltd., Shanghai, China) to detect the levels of inflammatory cytokines including interleukin-1β (IL-1β), interleukin-2 (IL-2), interleukin-10 (IL-10), and tumor necrosis factor-α (TNF-α) based on the manufacturer’s instructions. The optical density was measured using a Microplate Reader (Bio-Rad, Hercules, CA, USA) at 450 nm. The results were normalized to the protein concentration of each sample. Protein concentrations were quantified by a bicinchoninic acid protein Assay kit (Nanjing Jiancheng Bioengineering Institute, Nanjing, China). The contents of serum corticosterone and LPS were also determined by enzyme-linked immunosorbent assay.

### Real-time PCR analysis

The RT-PCR analysis was performed to determine gene expressions in terms of claudin-1, occludin, *ZO-1*, *TLR4*, *NF-κB*, and *MyD88* as previously described [39]. To be specific, total RNA was extracted from jejunal mucosa with the TRIzol reagent (Tiangen Biotech Co., Ltd., Beijing, China). The RT-PCR reactions were completed using SYBR Green on an ABI 6 flex real-time PCR instrument (Thermo Fisher Scientific, Waltham, MA, USA). Primer sequences were shown in Table S2 (Additional file 1). Relative mRNA expression levels were calculated according to the 2^−ΔΔCt^ method [40].

### Bacterial DNA extraction and 16S rRNA gene sequencing

A total of 300 mg cecal content samples were used to extract the total bacterial DNA using the E.Z.N.A Soil DNA Kit (Omega Bio-Tek, Norcross, GA, USA). The V3-V4 regions of the bacterial 16S rRNA gene were amplified using primer 338F (5′-ACTCCTACGGGAGGCAGCA-3′) and 806R (5′-ACTCCTACGGGAGGCAGCA-3′). The amplicons were sequenced at Shanghai Majorbio BioPharm Technology Co., Ltd., using the Illumina MiSeq platform, which was carried out according to the previous study [26].

### Bioinformatics analysis

Sequences were processed and taxonomy assigned using Quantitative Insights into Microbial Ecology 2 [41]. Amplicon sequence variants (ASVs) were determined with Dada2 and were classified at various taxonomic levels using the Silva 138 database as a reference template. α-diversity of microbiota was analyzed with Mothur (version 1.30.2). β-diversity was estimated using principal coordinate analysis (PCoA) accompanied by the analysis of similarities (ANOSIM). Using a non-parametric factorial Kruskal-Wallis sum-rank test to analyze the differences in the relative abundances of bacteria. Further bacteria as biomarkers were identified to distinguish microbiota of all groups by linear discriminant analysis combined effect size (LDA > 3, *P* < 0.05). the potential relationship between intestinal microbiota and phenotypes was conducted using Spearman correlation with the heatmap package. The co-occurrence of microbial communities was analyzed among the top 35 genera based on the significant Spearman correlations (|*R*| > 0.6, *P* < 0.05). Python package NetworkX was used to visualize the co-occurrence network.

### Measurement of SCFAs profile

A total of 300 mg cecal content samples were diluted with 1.5 mL of distilled water, and centrifugation was performed at 12,000 r/min for 10 min. Then 1 mL supernatant was added into a new centrifuge tube with 50 μL perchloric acid to centrifuge again under the same conditions for 3 h. The supernatant was removed and filtered through a membrane and then extracted with ethanol. The acetic acid, propionic acid, butyric acid, and lactic acid concentrations were detected using an ACQUITY UPLC I-Class system & VION IMS QTOF Mass spectrometer (Waters, Milford, MA, USA). The measurement of SCFAs was performed as previously described [42].

### Statistical analysis

Data analysis was performed using SAS Version 9.2 (SAS Institute Inc., Cary, NC, USA). The one-way ANOVA and Duncan’s multiple range test were used to evaluate the statistical significance. Statistical significance was declared at *P* < 0.05, while a tendency toward significance considered at 0.05 ≤ *P* < 0.10.

## Results

### Growth performance and litter quality and breast blister score

Compared with NC and OA group, decreased ADFI was found in the PC group during d 1 to 21 (*P* < 0.05). However, there were no differences in BW, ADG, and F/G among groups during d 1 to 21 (*P* < 0.05, Table 1). During d 22 to 42, the BW and ADG were decreased and were accompanied by a higher F/G in the PC group than that in the NC group. Simultaneously, higher BW, ADG, and decreased F/G were observed in the OA group compared with the PC group (Table 1). Moreover, the higher litter quality score in the PC group suggested that broiler diarrhea was caused by stocking density conditions on d 42 (*P* < 0.05, Additional file 1: Table S3). In addition, there were no significant differences in the breast blister score among groups (*P* > 0.05, Additional file 1: Table S3).

**Table 1:** Effects of high stocking density exposure on growth performance of broilers

Items	NC	PC	OA	SEM	*P*-value
d 0–21					
BW, kg	0.89	0.88	0.90	0.01	0.580
ADFI, g	52.96^a^	51.05^b^	52.79^a^	0.35	0.042
ADG, g	40.00	39.91	40.64	0.27	0.500
F/G	1.32	1.28	1.30	0.01	0.095
d 22–42					
BW, kg	2.41^ab^	2.29^b^	2.47^a^	0.03	0.014
ADFI, g	135.02	132.08	133.58	1.24	0.636
ADG, g	71.69	66.52	71.04	1.09	0.107
F/G	1.89	2.00	1.89	0.02	0.086

*NC* normal stocking density fed basal diet, *PC* high stocking density fed basal diets, *OA* high stocking density fed basal diets supplemented with organic acids, *BW* body weight, *ADG* average daily gain, *ADFI* average daily feed intake, *F/G* feed conversion ratio (feed:gain, g: g)

^a,b^Means within a row with no common superscript differ significantly (*n* = 12; *P* < 0.05)

### Stress and glucolipid metabolism parameters

To investigate whether HSD exposure can cause a stress response to broilers, stress, inflammation indicators (including corticosterone, LPS, and AST), and glucolipid metabolism in terms of GLU, TG, TC, HDL-C, and LDL-C were evaluated (Table 2). A higher level of corticosterone, LPS, and AST was observed in the PC group than that in the NC group (*P* < 0.05), which suggested HSD induced host stress. Nevertheless, there were no significant differences in the level of corticosterone, LPS, and AST between the NC and OA groups (*P* > 0.05). Furthermore, the significantly increased level of LDL accompanied by decreased level of GLU, TG, and the ratio of HDL-C to LDL-C were found in the PC group (*P* < 0.05) indicating HSD-induced disorders of glucolipid metabolism. Notably, no significant differences were observed in terms of GLU, LDL-C, and the ratio of HDL-C to LDL-C between the NC and OA groups (*P* > 0.05).

**Table 2:** Effects of high stocking density exposure on stress and glucolipid metabolism parameters

Items	NC	PC	OA	SEM	*P*-value
Corticosterone, ng/mL	21.88^b^	23.78^a^	22.54^b^	0.23	0.001
LPS, EU/L	10.74^b^	12.15^a^	11.06^b^	0.17	0.001
AST, U/L	361.20^b^	431.83^a^	408.30^ab^	12.49	0.059
GLU, mmol/L	10.14	9.25	9.68	0.17	0.101
TG, mmol/L	0.78^b^	0.53^a^	0.43^b^	0.04	0.001
TC, mmol/L	2.11	2.06	2.11	0.06	0.919
HDL-C, mmol/L	45.8^5^	44.52	44.76	0.88	0.814
LDL-C, mmol/L	2.27^b^	2.73^a^	2.15^b^	0.07	0.001
HDL-C / LDL-C	20.89^a^	16.85^b^	21.11^a^	0.77	0.035

*NC* normal stocking density fed basal diet, *PC* high stocking density fed basal diets, *OA* high stocking density fed basal diets supplemented with organic acids, *LPS* lipopolysaccharides, *AST* aspartate transaminase, *GLU* glucose, *TG* triglycerides, *TC* total cholesterol, *HDL-C* high-density lipoprotein cholesterol, *LDL-C* low-density lipoprotein cholesterol, *HDL-C/LDL-C* the ratio of high-density lipoprotein cholesterol to low-density lipoprotein cholesterol

^a,b^Means within a row with no common superscript differ significantly (*n* = 12; *P* < 0.05)

### Intestinal development and goblet cell analysis

**Fig. 1:**
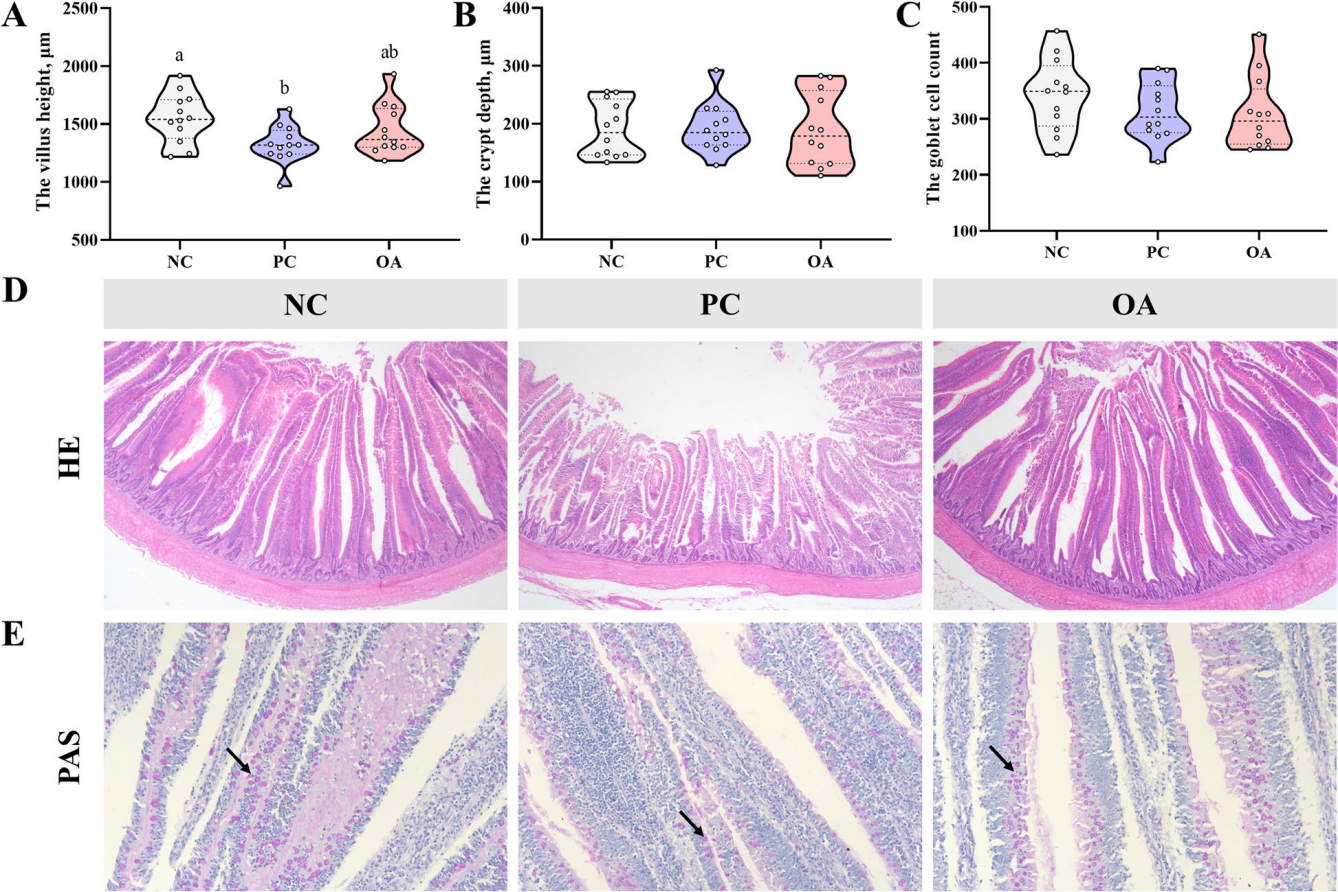
Intestinal morphological structure in broilers. **A, B** The morphological structure of jejunum; **C** The goblet cell count; **D** The morphological structure of jejunum, which was observed at 40 × magnification; **E** Distribution of goblet cells (purple cells with the black arrow) in the jejunum, which were observed at 200 × magnification; NC, normal stocking density fed basal diet; PC, high stocking density fed basal diets; OA, high stocking density fed basal diets supplemented with organic acids. ^a−b^Values at the same index with no common superscripts differ significantly (*P* < 0.05)

To further evaluate effects of dietary OA on the intestinal development of broilers with HSD exposure, we compared the intestinal relative weight, morphology, and goblet cell count, respectively. In Table S4 (Additional file 1), no significant differences in relative weight of intestine were found among the 3 groups on d 21 (*P* > 0.05). However, compared with the NC group on d 42, a significantly decreased relative weight of the duodenum, jejunum, and total intestine caused by HSD exposure was observed (*P* < 0.05). As shown in Fig. 1D, we could find that HSD exposure significantly decreased the jejunal villus height (VH) and broken villi of broilers in the PC group compared with the NC group on d 42 (*P* < 0.05). The results were statistically confirmed by the data shown in Fig. 1. Likewise, there were no significant differences in terms of jejunal VH, and crypt depth between the NC and OA groups (*P* > 0.05). Additionally, there were many purple-red goblet cells (black arrows) in the intestinal mucosa (Fig. 1E). However, no significant change in the goblet cell count was found among the 3 groups (*P* > 0.05).

### Inflammatory responses and the TLR4/NF-κB signaling pathway

**Fig. 2:**
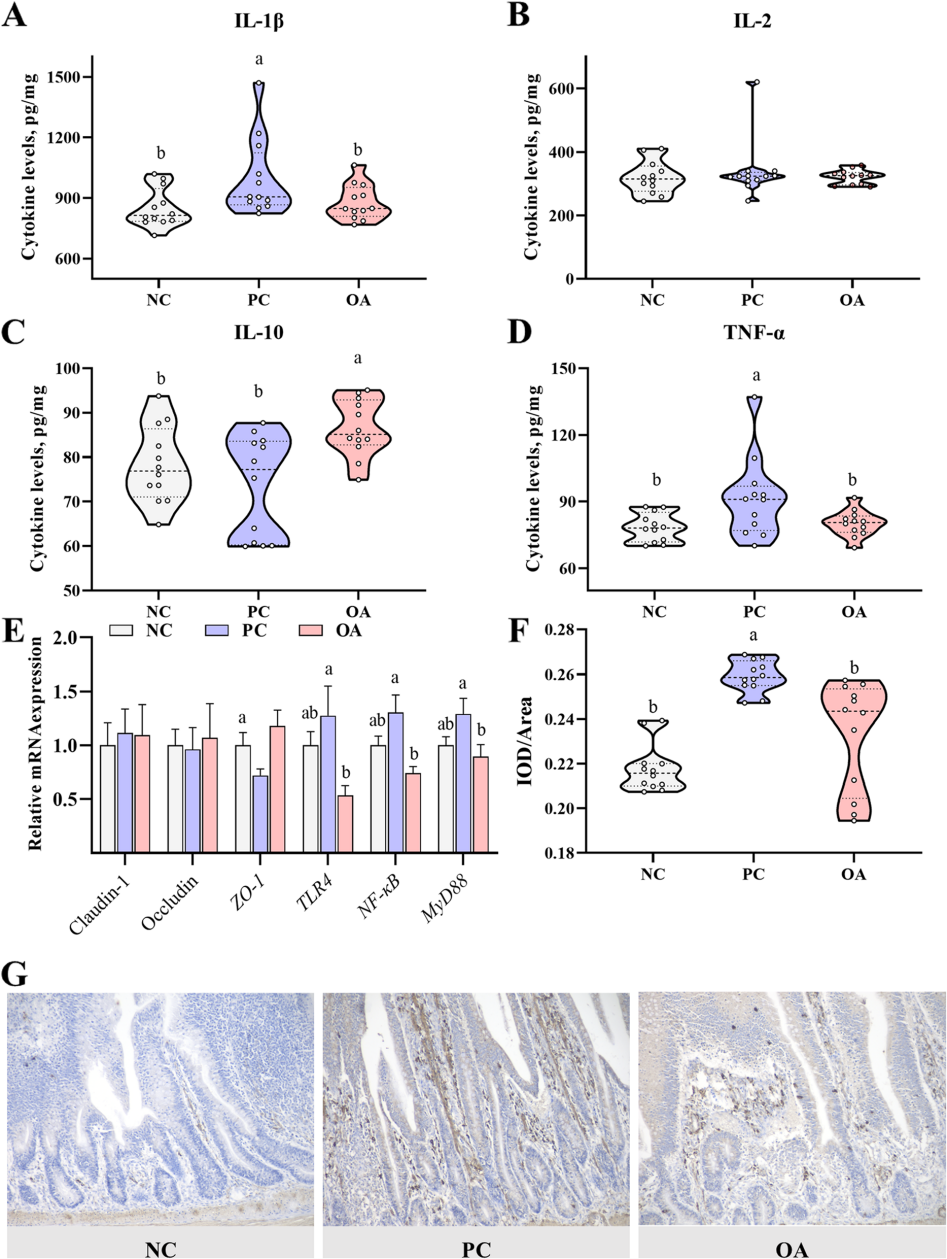
Effects of high stocking density exposure on intestinal barrier function and inflammatory response in broilers. **A** The level of interleukin 1β (IL-1β) in the jejunal mucosa; **B** The level of interleukin-2 (IL-2) in the jejunal mucosa; **C** The level of interleukin-10 (IL-10) in the jejunal mucosa; **D** The level of tumor necrosis factor-α (TNF-α) in the jejunal mucosa; **E** mRNA levels of tight junction and TLR4/NF-κB signaling pathway-related genes; **F** Quantitative analysis of the immunohistochemical expression of p-NF-кB; **G** The expression of p-NF-кB in jejunal tissues was examined by immunohistochemical analysis (200×, brown granules indicate positive reaction); NC, normal stocking density fed basal diet; PC, high stocking density fed basal diets; OA, high stocking density fed basal diets supplemented with organic acids; ^a−b^Values at the same index with no common superscripts differ significantly (*P* < 0.05)

To evaluate whether HSD stress caused poor intestinal morphology via inducing an inflammatory response and then damaging the intestinal barrier, we examined inflammatory cytokines and intestinal barrier markers (Fig. 2). Firstly, compared with the NC group, significantly increased levels of IL-1β and TNF-α were found in the PC group indicating HSD has induced intestinal inflammatory responses in broilers (*P* < 0.05, Fig. 2A, D). Interestingly, there were no differences in levels of IL-1β and TNF-α between the NC and OA groups (*P* > 0.05, Fig. 2A, D). Furthermore, the level of IL-10 in the OA group was significantly higher than that in NC and PC groups suggesting the anti-inflammatory effects of OA (*P* < 0.05, Fig. 2C). Compared with the NC group in terms of gene expression of tight junction proteins, the mRNA level of *ZO-1* was markedly down-regulated in the PC group (*P* < 0.05, Fig. 2E). Similarly, no significant difference in the mRNA level of *ZO-1* was observed between the NC and OA groups (*P* > 0.05, Fig. 2E).

Moreover, the related genes and proteins of the TLR4/NF-κB signaling pathway were determined via RT-PCR and immunohistochemistry staining analysis. As shown in Fig. 2E, the mRNA levels of *NF-κB* and *MyD88* were markedly up-regulated in the PC group and were accompanied by an up-regulated tendency in *TLR4* mRNA levels compared with those of the NC group (*P* < 0.05 or 0.05 ≤ *P* < 0.10). These results suggest that HSD stress caused intestinal inflammation via activating the TLR4/NF-κB signaling pathway in broilers. Notably, compared with the PC group, the mRNA levels of *TLR4*, *NF-κB* and *MyD88* were significantly down-regulated in the OA group (*P* < 0.05), which suggested OA ameliorated intestinal inflammation via inhibiting the TLR4/NF-κB signaling pathway. These results were further validated by immunohistochemical staining analysis. Positive brown granules were observed in the nucleus indicating that the TLR4/NF-κB was activated and expressed (Fig. 2G). Quantitative analysis of the immunohistochemistry confirmed that HSD stress significantly up-regulated the expression of p-NF-кB compared with the NC group (*P* < 0.05, Fig. 2F). Similarly, compared with the PC group, the expression of p-NF-кB was significantly down-regulated in the OA group (*P* < 0.05, Fig. 2F).

### Intestinal microbiota diversity

**Fig. 3:**
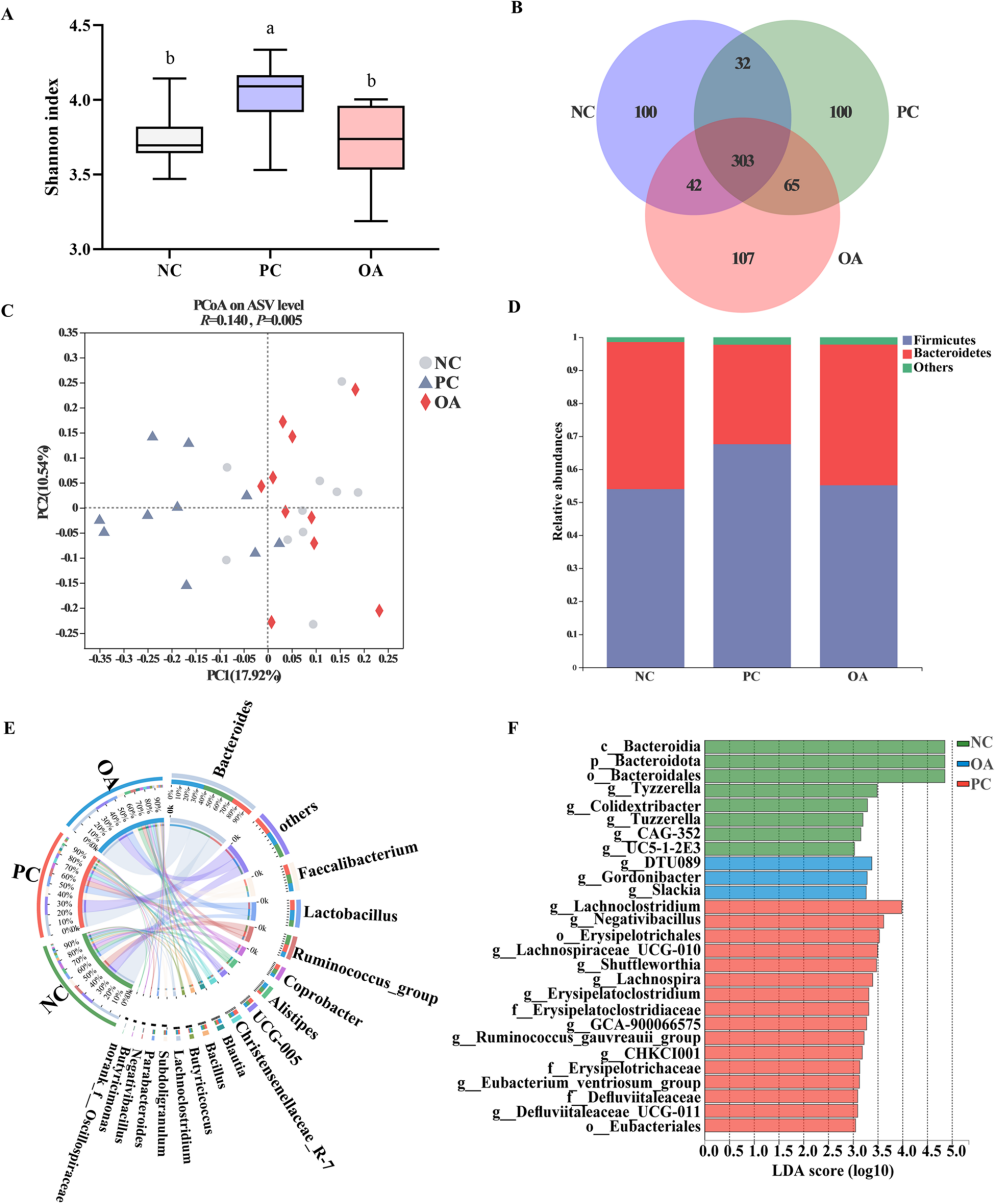
Microbial diversity analysis of broilers intestine. **A** Shannon index on the ASVs level; **B** A Venn diagram based on the ASVs level; **C** Principal coordinate analysis (PCoA) based on bray-curtis; **D** Microbial composition at the phylum level; **E** Microbial composition at the genus level; **F** Linear discriminant analysis effect size of intestinal microbiota (LDA > 3, *P* < 0.05); NC, normal stocking density fed basal diet; PC, high stocking density fed basal diets; OA, high stocking density fed basal diets supplemented with organic acids; ^a−b^Values at the same index with no common superscripts differ significantly (*P* < 0.05)

In the microbiome analysis, we obtained an average of 52,398 high-quality sequences after filtering. The significantly increasing Shannon index used to assess the microbiota α-diversity was observed in the PC group than that in other groups (*P* < 0.05, Fig. 3A). The β-diversity analysis was performed to evaluate the similarity of microbial profiles among 3 groups. As can be seen from Fig. 3C, all samples were mainly scattered into 2 clusters and samples from the PC group occupied distinct positions, which were clearly separated from NC and OA groups. ANOSIM analysis also indicated that the compositions of microbiota in the PC group were dissimilar from the NC group while OA restored the intestinal microbiota of broilers with HSD exposure and shaped it closer to the NC group (*R* = 0.140, *P* = 0.005). The Venn diagram revealed the difference in ASVs, and there were 100 and 107 unique ASVs in the PC and OA groups compared with the NC group, respectively (Fig. 3B). Additionally, Firmicutes and Bacteroidetes were major phylum species, and *Bacteroides*, *Faecalibacterium*, *Lactobacillus*, *Ruminococcus_torques_group*, *Coprobacter*, *Alistipes*, *UCG-005*, and *Christensenellaceae_R-7_group* were dominant genera across all groups (Fig. 3D, E).

Further, the changes in intestinal microbiota composition were analyzed. HSD stress significantly decreased the abundance of Bacteroidetes (*P* < 0.05, Fig. S1A, Additional file 1), which was accompanied by an increasing tendency in the abundance of Firmicutes (*P* = 0.078). At the genus level, a total of 24 differential bacteria were identified among all groups, with the abundance of *Christensenellaceae_R-7_group*, *Lachnoclostridium*, *Negativibacillus*, *Erysipelatoclostridium*, *Eubacterium_hallii_group*, *GCA-900066575*, *Ruminococcus_gauvreauii_group*, *Defluviitaleaceae_UCG-011*, and *Merdibacter* were significantly increasing in the PC group (*P* < 0.05, Fig. S1B, Additional file 1). However, the abundance of *Bacteroides*, *Ruminococcus_torques_group*, and *Anaerotruncus* showed a decreasing trend in the PC group compared with NC and OA groups (0.05 ≤ *P* < 0.10). Furthermore, bacteria as biomarkers were identified to distinguish microbiota of all groups by the LEfSe analysis. As shown in Fig. 3F, microbes in the PC group were enriched with *Lachnoclostridium*, *Lachnospiraceae_UCG-010*, *Shuttleworthia*, *Lachnospira*, *GCA-900066575*, *Eubacterium_ventriosum_group*, and *CHKCI001*, which all belong to the Lachnospiraceae. Besides, we found that Defluviitaleaceae (*Defluviitaleaceae_UCG-011*), Erysipelatoclostridiaceae (*Erysipelatoclostridium*), Ruminococcaceae (*Negativibacillus*) were linked to the increased relative abundance in the PC group, whereas the Eggerthellaceae (*Gordonibacter* and *Slackia*) and Ruminococcaceae (*DTU089*) were characterized to be enriched in the OA group.

### Correlations between intestinal microbiota and phenotypes

**Fig. 4:**
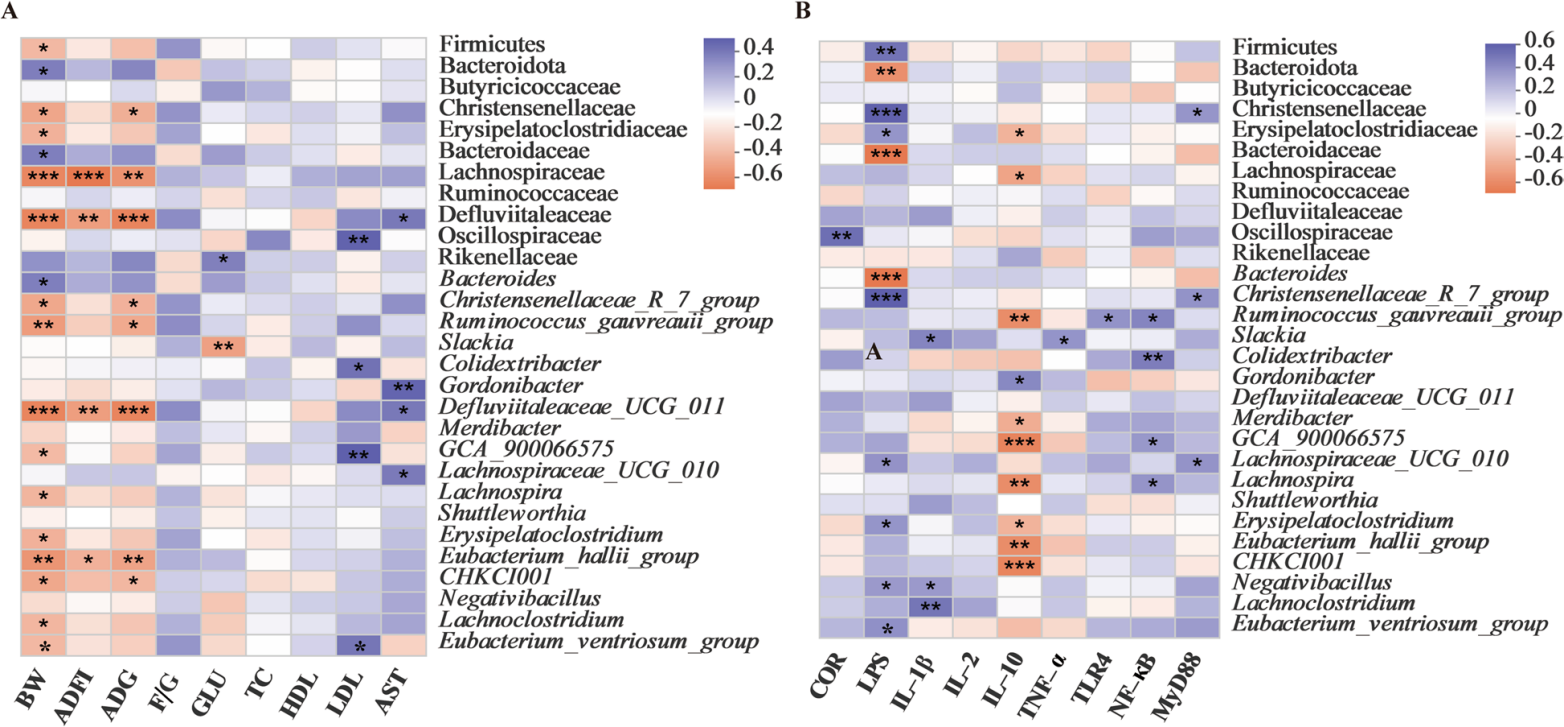
Spearman’s correlation analysis between the abundances of intestinal microbiota and phenotypes. **A** The correlation analysis between intestinal microbiota and growth performance and serum biochemical parameters. **B** The correlation analysis between intestinal microbiota and intestinal inflammatory indicators. Blue represents a positive correlation and red represents a negative correlation. Significant correlations are noted by 0.01 < *P* ≤ 0.05*, 0.001 < *P* ≤ 0.01**, *P* ≤ 0.001***

To explore the microbiota associated with growth performance and intestinal inflammation, correlations between the abundance of the bacteria and phenotypes were analyzed (Fig. 4). The abundance of phylum Firmicutes mainly including Christensenellaceae, Erysipelatoclostridiaceae, Lachnospiraceae, and Defluviitaleaceae showed significant negative correlations with the growth performance and positive correlations with serum LPS, whereas the abundance of phylum Bacteroidetes including Bacteroidaceae were positively correlated with the growth performance and negatively correlated with serum LPS, respectively. In addition, the abundance of Defluviitaleaceae, Oscillospiraceae, Rikenellaceae, and Christensenellaceae were positively correlated with the level of serum AST, LDL, CLU, and the expression of *MyD88*, respectively. However, the abundance of Erysipelatoclostridiaceae and Lachnospiraceae showed significant negative correlations with the level of IL-10 in the intestinal mucosa. Likewise, numerous significant correlations were also found at the genus level of microbiota with the growth performance, serum parameters, inflammatory cytokines, and intestinal barrier markers.

### Intestinal microbiota co-occurrence network analysis

**Fig. 5:**
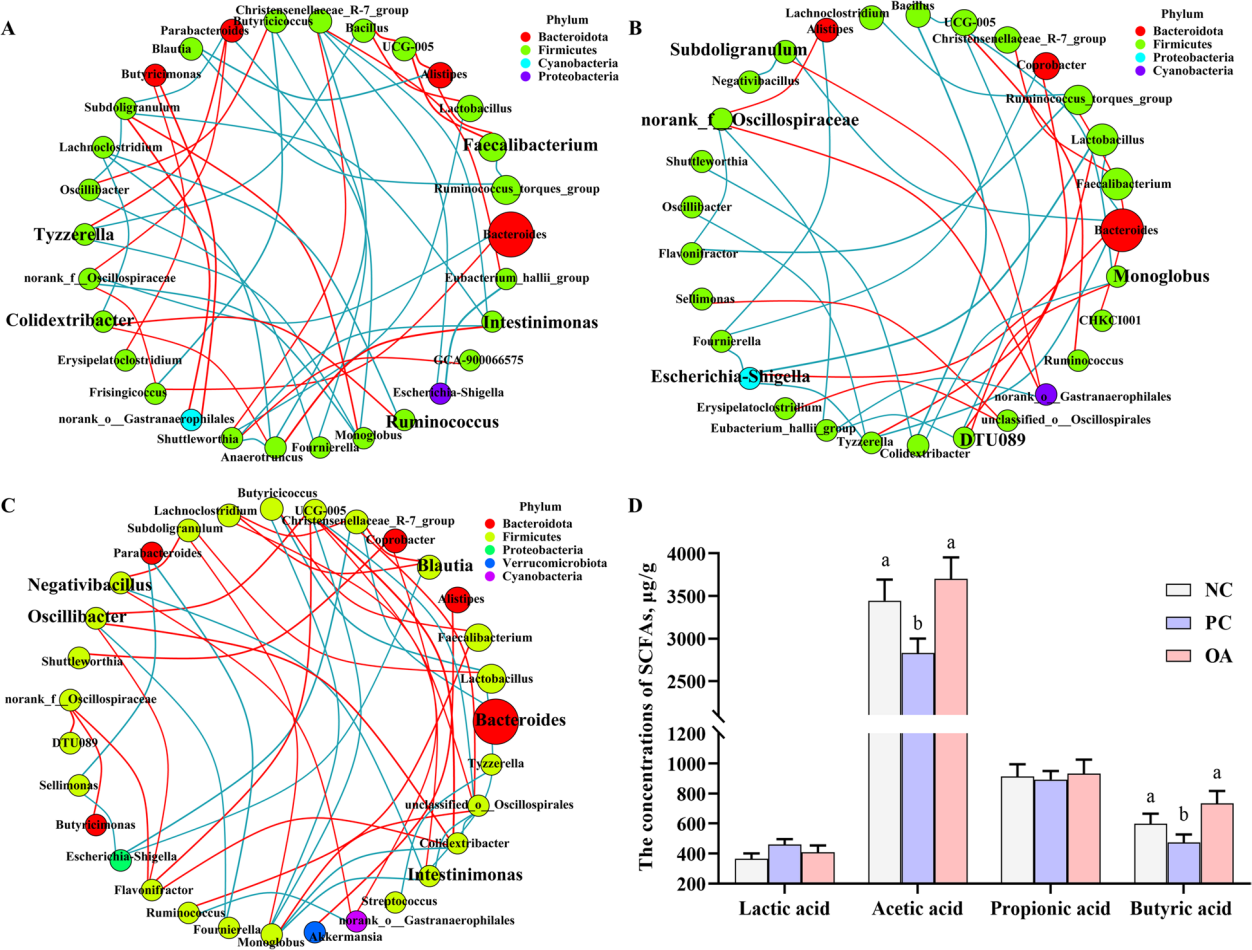
Microbial communities co-occurrence network and short-chain fatty acids analysis. **A** The intestinal microbiota in the NC group (normal stocking density fed basal diet); **B** The intestinal microbiota in the PC group (high stocking density fed basal diets); **C** The intestinal microbiota in the OA group (high stocking density fed basal diets supplemented with organic acids); The size of nodes shows the abundance of the species. Red and green lines represent positive and negative correlations, respectively (**D**) Concentrations of cecal short-chain fatty acids in broilers. ^a−b^Values at the same index with no common superscripts differ significantly (*P* < 0.05)

Furthermore, the co-occurrence of microbial communities was analyzed to explore the co-existence and interaction of species induced by the HSD stress and dietary OA. The correlation network analysis showed 44, 36, and 47 significant correlations in NC, PC, and OA groups, respectively (Fig. 5A-C). There was a difference in the proportion of positive and negative links in the PC group (33.3%:66.7%) compared with NC (47.8%:52.3%) and OA (53.2%:46.8%) groups. Moreover, *DTU089*, *Escherichia-Shigella*, *UCG-005*, *norank_f__Oscillospiraceae*, *Subdoligranulum*, and *Monoglobus* were selected as the keystone genera in the PC group based on degree centrality, closeness centrality, and betweenness centrality scores. However, *Colidextribacter*, *Intestinimonas*, *Ruminococcus*, *Christensenellaceae_R-7_group*, *Tyzzerella*, and *Faecalibacterium* were selected as the keystone genera in the NC group. Likewise, *Christensenellaceae_R-7_group* and *Intestinimonas* were also identified as the keystone genera in the OA group, besides *Negativibacillus*, *Oscillibacter*, *Blautia*, and *Bacteroides*.

### Intestinal SCFAs profiles and regression analysis

**Fig. 6:**
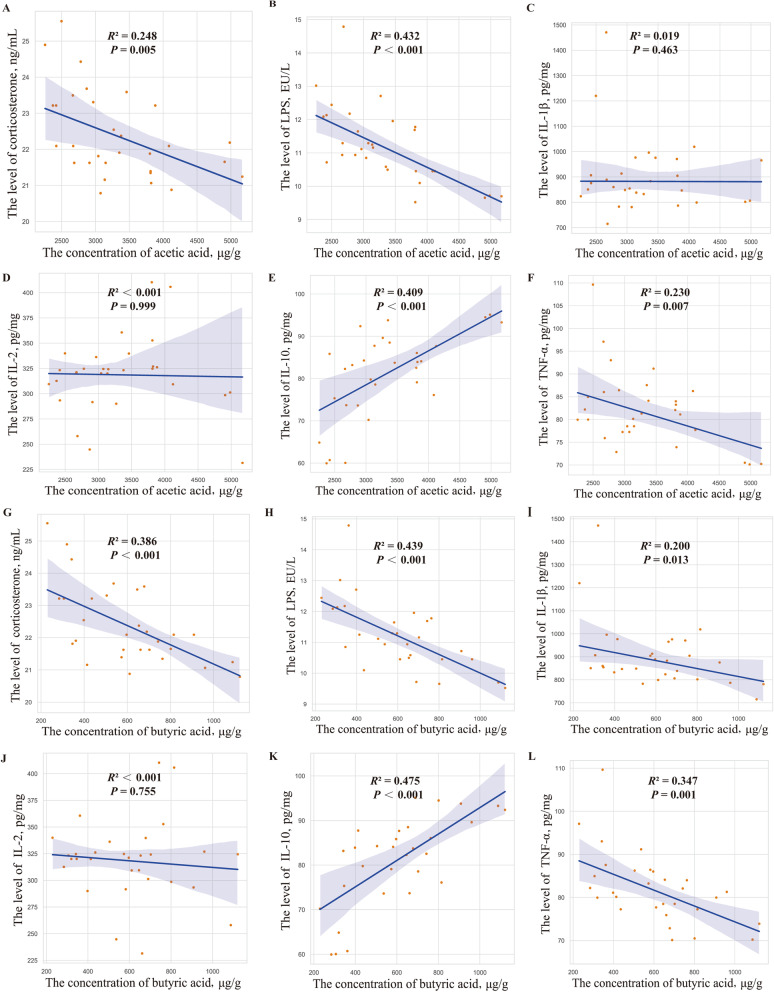
The regression analysis between the short-chain fatty acids and intestinal inflammatory parameters. **A-F** The acetic acid concentration and its association with corticosterone, lipopolysaccharides (LPS), interleukin-1β (IL-1β), interleukin-2 (IL-2), interleukin-10 (IL-10), and tumor necrosis factor-α (TNF-α); **G-L** The butyric acid concentration and its association with corticosterone, LPS, IL-1β, IL-2, IL-10, and TNF-α

Differences in the co-occurrence of microbial communities suggested that the bacteria within them perform different functions. Therefore, major carbohydrate fermentation products of gut microbiota were detected and quantified here to evaluate microbial activity (Fig. 5D). Compared with the NC group, significantly decreased contents of acetic and butyric acids were found in the PC group in response to HSD (*P* < 0.05). However, there were no significant differences in the content of acetic and butyric acids between the NC and OA groups (*P* > 0.05). To further confirm the role of microbiota in response to intestinal inflammation, we conducted the regression analysis between the concentration of significantly different SCFAs and levels of stress and inflammatory cytokines (Fig. 6). Significant linear negative correlations were detected between the concentration of acetic acid and levels of corticosterone, LPS, and TNF-α, which was accompanied by a linear positive correlation with the level of IL-10. Meanwhile, significant linear correlations were observed between the concentration of butyric acid and levels of inflammatory cytokines except for IL-2.

## Discussion

Stocking broilers under a high density is one of the strategies to optimize the area of the cage and hence achieve a high level of efficiency in broiler production. However, accumulating studies have reported that HSD could induce adverse effects on broilers in terms of growth performance and physiological conditions [4–6]. Consistent with previous studies, the compromised BW, ADG, and feed conversion ratio of broilers were induced by the HSD conditions in the present study. As one of the indicators of physiological stress conditions in chickens [43], the level of corticosterone is also elevated under the HSD conditions. In general, the HSD stress activates the hypothalamic-pituitary-adrenal axis to trigger corticosterone secretion [7]. Therefore, the above results suggest that a stress model of the HSD was established successfully. The negative effect of HSD on growth performance of broilers is attributable to the competition for feeding and drinking water, elevated room temperature, and increased contents of moisture and ammonia in litter induced by overcrowding, consequently impairing feed digestibility and nutrient utilization [2, 44]. Additionally, with regard particularly to the physiological condition, it is a crucial factor to ensure the healthy growth of broilers, which is also easily influenced by the HSD stress [11]. Consistent with previous studies, disorders of glucolipid metabolism, especially changed concentration in terms of GLU, TG, LDL-C, and the ratio of HDL-C to LDL-C of broilers exposed to the HSD stress were also observed in the current study [10, 11]. Meanwhile, broilers had a higher level of serum AST under the HSD conditions, which also confirm hepatocellular injury with metabolic disorders. In addition, the increased level of corticosterone induced by the HSD stress further decreased the level of the thyroxine hormone, resulting in metabolic disorders in broilers [8, 9]. Therefore, disturbances in glucolipid metabolism due to the HSD stress could partially explain the poor growth performance in broilers.

Numerous studies have reported the beneficial effect of OA on growth performance in poultry [26–28]. This study also found that organic acids improved compromised growth performance induced by HSD stress. Generally, OA can reduce the intestinal lumen pH and increase digestive enzyme activity to improve the apparent digestibility [29, 30], indicating the important role of OA in maintaining intestinal health. Unfavorable alterations in growth performance and glycolipid metabolism suggested that the intestine has been damaged in the PC group. The normal intestinal morphology and complete intestinal mucosal barrier play an important role in maintaining normal metabolism and immune responses [45, 46]. In particular, tight junction proteins including claudin, occludin, and zonula occludens families maintain the physical barrier of the intestinal epithelium to determine the selective permeability and enhance the intestinal defensive function [47, 48]. However, the tight junction proteins are vulnerable to the exposure of external stressors, which cause local or systemic inflammation [49]. In the present study, HSD stress impaired intestinal development and intestinal barrier in terms of the decreased jejunal VH and the mRNA level of *ZO-1*. The intestinal permeability will be increased once the intestinal barrier is damaged, which was often accompanied by an increased LPS level [5, 15]. In agreement, our results also revealed dietary OA could improve the impaired intestinal barrier function [26, 50], which was proven by the reduced LPS level. As a marker of intestinal barrier damage, LPS can bind to TLR4 and stimulate the downstream signaling molecule *MyD88* and *NF-κB p65* to mediate inflammatory responses [16]. Subsequently, pro-inflammatory cytokines IL-1β and TNF-α produced by inflammatory responses rearranged tight junction proteins to damage intestinal barrier function [51]. Notably, inhibition of the TLR4/NF-κB signaling pathway and decreased pro-inflammatory cytokines IL-1β and TNF-α levels were found in the OA group. Therefore, the current study suggests that dietary OA can inhibit the TLR4/NF-κB signaling pathway to improve the HDS stress-induced intestinal inflammation in broilers.

It is important to note that the LPS is composed of lipids and polysaccharides in the outer membrane of the cell wall of Gram-negative bacteria [17]. Considering that LPS release is significantly enhanced when the bacteria are lysed [52], the change in the serum LPS level indicates a dramatic shift in intestinal microbiota [53]. It has generally been known that the key role of the intestinal microbiota is involved in maintaining intestinal barrier function and energy metabolism [54]. Previous studies have found alterations in the intestinal microbiota of broilers induced by HSD stress [4, 18, 19]. Likewise, the present study revealed HSD stress significantly changed the α and β diversity of the intestinal microbiota in broilers. The increased corticosterone level induced by the stress could cause the dysbiosis of gut microbiota, in terms of decreased Bacteroides abundance, increased Firmicutes abundance, and α diversity [55–57]. In agreement, this study further revealed that Lachnospiraceae, Defluviitaleaceae, Erysipelatoclostridiaceae, and Ruminococcaceae were identified as biomarkers in broilers exposed to HSD stress. Additionally, HSD stress significantly decreased microbial correlations while increased competitive and exploitative interactions in microbial communities, which could also disturb the balance of microbial ecosystems and subsequently caused negative effects on intestinal health [39]. However, whether the ameliorative effects of OA on the HDS stress-induced inflammatory responses are related to the intestinal microbiota of broilers remains elusive.

OA can alter the intestinal microbiota of broilers due to its antimicrobial action via perforating semipermeable peptidoglycan or phospholipid membrane, then the dissociation and release of hydrogen ions reduce the pH to induce bacterial collapse [26, 31, 32]. In this study, OA restored the intestinal microbiota of broilers with HSD exposure and reshape the microbial compositions and interactions. In particular, OA significantly increased the abundance of Bacteroidetes and microbial symbiosis, while the abundance of Firmicutes and competitive interactions in the intestinal microbiota were decreased. Notably, Christensenellaceae, Erysipelatoclostridiaceae, Lachnospiraceae, and Defluviitaleaceae belonging to phylum Firmicutes showed negative effects on the growth performance and intestinal barrier function, whereas Bacteroidaceae belonging to phylum Bacteroidetes showed positive effects. Growing evidence has demonstrated that Bacteroidetes play important roles in improving the balance of intestinal microbiota, fermenting carbohydrates, and inhibiting pathogen colonization [58, 59]. The increased ratio of Firmicutes to Bacteroidetes was found to be associated with intestinal microbiota dysbiosis and metabolic diseases [55, 60]. LPS is a major component of the outer membrane of the cell wall in Gram-negative bacteria and plays an important role in triggering intestinal inflammatory responses [16]. The increased LPS content may be due to competitive exclusion induced by excessive Firmicutes, leading to Bacteroidetes lysis and thus the release of LPS in broilers exposed to HSD stress [52]. Therefore, the re-establishment of microbial compositions and interactions suggest that intestinal microbiota play critical roles in ameliorative effects of OA on the HDS stress-induced inflammatory responses of broilers in the current study.

To further investigate the potential approach to intestinal microbiota involved in regulating intestinal inflammatory responses, SCFAs as the major carbohydrate fermentation products of microbiota were detected. It is worth noting that the improvement of HSD stress-induced adverse results was accompanied by a significant increase in contents of acetic and butyric acids. The available body of fact indicated that acetic acid is produced mainly by Bacteroidetes [61], which is consistent with the elevated Bacteroidetes and acetic acid contents in NC and OA groups. Additionally, butyric acid did not significantly increase in broilers exposed to HSD stress in the current study although it could be produced by Firmicutes. This result was consistent with the previous study reporting microbiota dysbiosis could reduce microbial activity and SCFA production [62]. The butyric acid synthesis has two primary pathways including the butyrate kinase pathway and the butyryl-CoA:acetate-CoA-transferase pathway in intestinal microbiota [63, 64]. It has been demonstrated that the butyryl-CoA:acetate-CoA-transferase pathway is the main process for the biosynthesis of butyric acid and acetic acid as a substrate involves this process [64, 65]. Therefore, in the present study, the increased butyric acid primarily depends on the production of acetic acid by Bacteroidetes to induce the synthesis of butyric acid rather than increasing the production of butyric acid by Firmicutes directly. Apart from crucial roles as energy sources for intestinal epithelial cells [66], growing evidence has demonstrated that SCFAs can inhibit the activation of the TLR4/NF-κB signaling pathway and contribute to the suppressed generation of pro-inflammatory cytokines [23–25]. Consistently, significant linear negative correlations were observed between SCFAs (acetic and butyric acids) and stress and pro-inflammatory cytokines, which was accompanied by a linear positive correlation with anti-inflammatory cytokines in the present study. Therefore, the HSD stress-induced intestinal microbiota dysbiosis could promote the production of pro-inflammatory cytokines via producing excess LPS to activate the TLR4/NF-κB signaling pathway. Accordingly, dietary OA can restore the gut microbial balance and elevate SCFAs production to inhibit the TLR4/NF-κB signaling pathway and ameliorate HSD stress-induced intestinal inflammation in broilers.

## Conclusions

In summary, dietary OA ameliorated intestinal inflammation and growth performance of broilers through restoring compositions and interactions in disorder gut microbiota induced by HSD and elevating SCFAs production to inhibit the TLR4/NF-κB signaling pathway. These findings demonstrated the critical role of intestinal microbiota in mediating the HSD-induced inflammatory responses, contributing to exploring nutritional strategies to alleviate HSD-induced stress in animals.

## Supplementary Information

**Additional file 1: Table S1.** Basal diet composition and nutrient level (air dry basis). **Table S2.** Primer sequences of target and reference genes. **Table S3.** Effects of high stocking density exposure on litter quality and breast blister score. **Table S4.** The effect of high stocking density conditions on intestine development of broilers. **Fig. S1.** Significantly differential bacteria at phylum and genus level.

## Data Availability

Not applicable.

## Abbreviations

ADFI: Average daily feed intake
ADG: Average daily gain
ANOSIM: Analysis of similarities
AST: Aspartate transaminase
ASVs: Amplicon sequence variants
BW: Body weight
GLU: Glucose
HDL-C: High-density lipoprotein cholesterol
HSD: High stocking density
IL-1β: Interleukin-1β
IL-2: Interleukin-2
IL-10: Interleukin-10
LDA: Linear discriminant analysis
LDL-C: Low-density lipoprotein cholesterol
LPS: Lipopolysaccharides
OA: Organic acids
PCoA: Principal coordinate analysis
p-NF-κB: Phosphorylation of NF-κB
qRT-PCR: Quantitative Real-time PCR
SCFAs: Short-chain fatty acids
TC: Total cholesterol
TG: Triglycerides
TLR4: Toll-like receptor 4
TNF-α: Tumor necrosis factor-α
VH: Villus height
ZO-1: Zonula occludens-1
