# Supplementary material for: Dietary organic acids ameliorate high stocking density stress-induced intestinal inflammation through the restoration of intestinal microbiota in broilers
Source: J Anim Sci Biotechnol. 2022 Nov 14;13:124. doi: 10.1186/s40104-022-00776-2 (PMC9661804; doi:10.1186/s40104-022-00776-2)
Supplement: Supplementary file 1 — Additional file 1: Table S1. Basal diet composition and nutrient level (air dry basis). Table S2. Primer sequences of target and reference genes. Table S3. Effects of high stocking density exposure on litter quality and breast blister score. Table S4. The effect of high stocking density conditions on intestine development of broilers. Fig. S1. Significantly differential bacteria at phylum and genus level. [file 40104_2022_776_MOESM1_ESM.docx]

**Table S1** Basal diet composition and nutrient level (air dry basis)

| Item | Starting phase (d 1–21) | Growing phase (d 22–42) |
| --- | --- | --- |
| Ingredients, % |  |  |
| Corn | 49.05 | 46.60 |
| Soybean meal | 33.55 | 28.98 |
| Wheat | 10.00 | 15.00 |
| Soybean oil | 2.42 | 5.21 |
| Dicalcium phosphate | 1.97 | 2.00 |
| Limestone | 1.2 | 0.90 |
| Salt | 0.15 | 0.21 |
| *DL*-Methionine | 0.34 | 0.19 |
| *L*-lysine-HCl | 0.33 | 0.09 |
| Threonine | 0.1 | 0.1 |
| Vitamin premix^a^ | 0.02 | 0.02 |
| Mineral premix^b^ | 0.20 | 0.20 |
| Choline chloride (50%) | 0.10 | 0.10 |
| Dicarbonate | 0.26 | 0.19 |
| Corn starch | 0.30 | 0.20 |
| Phytase (5,000 U/g) | 0.01 | 0.01 |
| Total | 100.00 | 100.00 |
| Nutrient levels |  |  |
| ME, kcal/kg | 2950 | 3150 |
| Crude protein, % | 22.01 | 20.00 |
| Calcium, % | 1.00 | 0.90 |
| Available phosphorus, % | 0.46 | 0.46 |
| Lysine, % | 1.27 | 0.97 |
| Methionine, % | 0.61 | 0.44 |
| Methionine + cystine, % | 0.94 | 0.76 |

^a^The vitamin premix supplied the following per kg of complete feed: vitamin A, 12 500 IU; vitamin D_3_, 2500 IU; vitamin K_3_, 2.65 mg; vitamin B_1_, 2 mg; vitamin B_2_, 6 mg; vitamin B_12_, 0.025 mg; vitamin E, 30 IU; biotin,0.0325 mg; folic acid, 1.25 mg; pantothenic acid, 12 mg; niacin, 50 mg

^b^The mineral premix supplied the following per kg of complete feed: Cu, 8 mg; Zn, 75 mg; Fe, 80 mg; Mn, 100 mg; I, 0.35 mg, Se, 0.15 mg

**Table S2** Primer sequences of target and reference genes

| Gene name | Forward primer (5' to 3') | Reverse primer (5' to 3') | GenBank number |
| --- | --- | --- | --- |
| Claudin-1 | CATACTCCTGGGTCTGGTTGGT | GACAGCCATCCGCATCTTCT | NM_001013611.2 |
| Occludin | TCATCGCCTCCATCGTCTAC | TCTTACTGCGCGTCTTCTGG | NM 205128.1 |
| *ZO-1* | CTTCAGGTGTTTCTCTTCCTCCTC | CTGTGGTTTCATGGCTGGATC | XM_015278981.2 |
| *TLR4* | GTCTCTCCTTCCTTACCTGCTGTTC | AGGAGGAGAAAGACAGGGTAGGTG | NM_001030693.1 |
| *NF-κB* | GCACAACGCCTCTTCACATA | GGCTCAAAGTTCTCAACGTG | NM_205134.1 |
| *MyD88* | ATCCGGACACTAGAGGGAGG | GGCAGAGCTCAGTGTCCATT | NM_001030962.1 |
| *β-actin* | ATCCGGACCCTCCATTGTC | AGCCATGCCAATCTCGTCTT | NM 205518.1 |

**Table S3** Effects of high stocking density exposure on litter quality and breast blister score

| Items | NC | PC | OA | SEM | *P*-value |
| --- | --- | --- | --- | --- | --- |
| Day 0–21 | | | | | |
| Litter quality | 1.17 | 1.33 | 1.17 | 0.07 | 0.549 |
| Breast blister | 1.42 | 1.25 | 1.25 | 0.10 | 0.728 |
| Day 22–42 | | | | | |
| Litter quality | 1.42^b^ | 2.42^a^ | 1.42^b^ | 0.12 | 0.001 |
| Breast blister | 1.75 | 1.75 | 1.58 | 0.15 | 0.883 |

^a,b^Means within a row with no common superscript differ significantly (*n* = 12; *P* < 0.05)

*NC*, normal stocking density fed basal diet; *PC*, high stocking density fed basal diets; *OA*, high stocking density fed basal diets supplemented with organic acids

**Table S4** The effect of high stocking density conditions on intestine development of broilers

| Items | NC | PC | OA | SEM | *P*-value |
| --- | --- | --- | --- | --- | --- |
| The relative weight on day 21, % | | | | | |
| Duodenum | 1.352 | 1.258 | 1.423 | 0.032 | 0.114 |
| Jejunum | 1.902 | 1.727 | 1.821 | 0.041 | 0.227 |
| Ileum | 1.419 | 1.398 | 1.379 | 0.039 | 0.920 |
| Total intestine | 4.675 | 4.382 | 4.622 | 0.083 | 0.314 |
| The relative weight on day 42, % | | | | | |
| Duodenum | 0.837^a^ | 0.687^b^ | 0.716^b^ | 0.019 | 0.001 |
| Jejunum | 1.358^a^ | 1.116^b^ | 1.158^b^ | 0.031 | 0.002 |
| Ileum | 0.998 | 0.970 | 0.957 | 0.029 | 0.851 |
| Total intestine | 3.192^a^ | 2.773^b^ | 2.830^b^ | 0.062 | 0.008 |

^a,b^Means within a row with no common superscript differ significantly (*n* = 12; *P* < 0.05)

*NC*, normal stocking density fed basal diet; *PC*, high stocking density fed basal diets; *OA*, high stocking density fed basal diets supplemented with organic acids


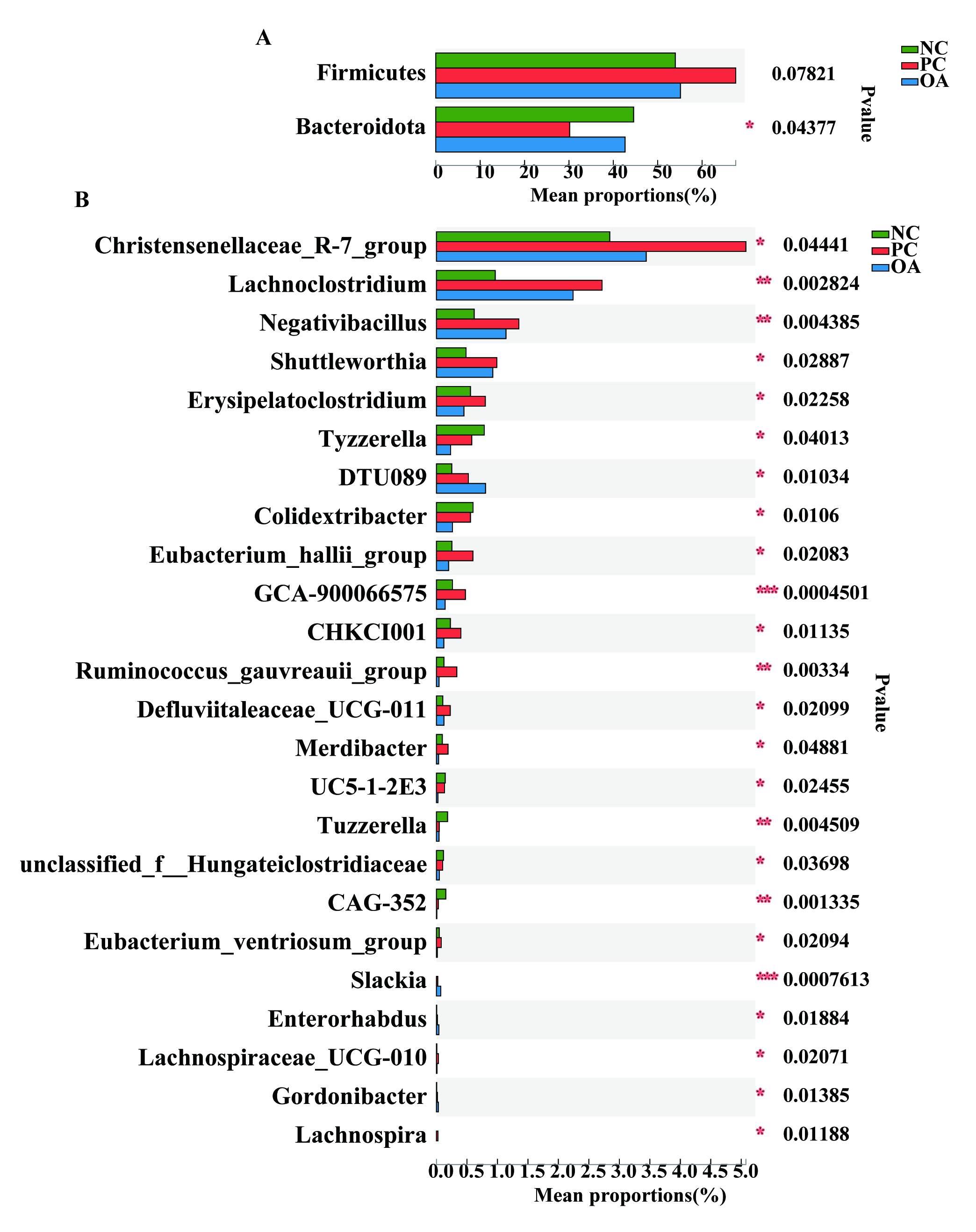


**Fig. S1** Significantly differential bacteria at phylum and genus level. (**A**) phylum level; (**B**) genus level; NC, normal stocking density fed basal diet; PC, high stocking density fed basal diets; OA, high stocking density fed basal diets supplemented with organic acids; “^*^” indicates statistically significant difference (^*^*P* < 0.05, ^**^*P* < 0.01 and ^***^*P* < 0.001)
